# Chicory: Understanding the Effects and Effectors of This Functional Food

**DOI:** 10.3390/nu14050957

**Published:** 2022-02-23

**Authors:** Céline L. Pouille, Souad Ouaza, Elise Roels, Josette Behra, Melissa Tourret, Roland Molinié, Jean-Xavier Fontaine, David Mathiron, David Gagneul, Bernard Taminiau, Georges Daube, Rozenn Ravallec, Caroline Rambaud, Jean-Louis Hilbert, Benoit Cudennec, Anca Lucau-Danila

**Affiliations:** 1 UMR Transfrontalière BioEcoAgro N° 1158, Univ. Lille, INRAE, Univ. Liège, UPJV, JUNIA, Univ. Artois, Univ. Littoral Côte d’Opale, ICV, SFR Condorcet FR CNRS 3417—Institut Charles Viollette, 59655 Villeneuve d’Ascq, France; celine.pouille.etu@univ-lille.fr (C.L.P.); souad.ouaza.etu@univ-lille.fr (S.O.); elise.roels@oniris-nantes.fr (E.R.); josette.behra@univ-lille.fr (J.B.); melissa.tourret@univ-lille.fr (M.T.); david.gagneul@univ-lille.fr (D.G.); rozenn.ravallec@univ-lille.fr (R.R.); caroline.rambaud@univ-lille.fr (C.R.); jean-louis.hilbert@univ-lille.fr (J.-L.H.); benoit.cudennec@univ-lille.fr (B.C.); 2 Joint Laboratory CHIC41H University of Lille-Florimond-Desprez, Cité scientifique, 59655 Villeneuve d’Ascq, France; 3 UMR Transfontalière BioEcoAgro N° 1158, Univ. Lille, INRAE, Univ. Liège, UPJV, JUNIA, Univ. Artois, Univ. Littoral Côte d’Opale, ICV, SFR Condorcet FR CNRS 3417—BIOlogie des Plantes et Innovation (BIOPI), 80025 Amiens, France; roland.molinie@u-picardie.fr (R.M.); jean-xavier.fontaine@u-picardie.fr (J.-X.F.); 4 Plateforme Analytique UFR des Sciences, UPJV, Bâtiment Serres-Transfert Rue Dallery-Passage du Sourire d’Avril, 80039 Amiens, France; david.mathiron@u-picardie.fr; 5 Department of Food Sciences–Microbiology, FARAH, University of Liege, 4000 Liege, Belgium; bernard.taminiau@uliege.be (B.T.); georges.daube@uliege.be (G.D.)

**Keywords:** chicory, transcriptomics, hormone assay, gut microbiota, in vitro apoptosis, in vitro pro-inflammatory cytokines

## Abstract

Industrial chicory has been the subject of numerous studies, most of which provide clinical observations on its health effects. Whether it is the roasted root, the flour obtained from the roots or the different classes of molecules that enter into the composition of this plant, understanding the molecular mechanisms of action on the human organism remains incomplete. In this study, we were interested in three molecules or classes of molecules present in chicory root: fructose, chlorogenic acids, and sesquiterpene lactones. We conducted experiments on the murine model and performed a nutrigenomic analysis, a metabolic hormone assay and a gut microbiota analysis, associated with in vitro observations for different responses. We have highlighted a large number of effects of all these classes of molecules that suggest a pro-apoptotic activity, an anti-inflammatory, antimicrobial, antioxidant, hypolipidemic and hypoglycemic effect and also an important role in appetite regulation. A significant prebiotic activity was also identified. Fructose seems to be the most involved in these activities, contributing to approximately 83% of recorded responses, but the other classes of tested molecules have shown a specific role for these different effects, with an estimated contribution of 23–24%.

## 1. Introduction

Industrial chicory (*Cichorium intybus* var. *sativum*) has been analyzed for its various dietary and medicinal effects. A substantial amount of clinical evidence depicts chicory to be anti-diabetic, immunomodulatory, anti-tumor, antioxidant, anthelmintic, and prebiotic. In addition, chicory has been shown to promote good digestion, to regulate appetite, and to decrease the risk of gastrointestinal diseases [1].

The chicory root, processed into flour and used as an ingredient for pastries, has been proposed as a functional food; potential mechanisms by which the chicory acts on cancer prevention, antibacterial and antiviral defense, hypoglycemic, hypolipidemic and antioxidant effects have been identified [2]. Among the major compounds of chicory root, inulin has been the subject of multiple studies concerning its prebiotic effect [2,3], but less is known about the specific mechanisms of other molecules that enter the composition of roots. The hydrolysis products of inulin, fructose and oligofructose, have been described to have multiple beneficial effects on bowel functions [4]. Chlorogenic acids (CGA) have been described to improve the insulin sensitivity in diabetic rats [5] and were also associated with major in vivo antioxidant properties [6] and an in vitro antibacterial activity [7]. Sesquiterpene lactones (STL), from the chicory leaves and roots, are known for their strong inhibitory effect on some nematodes infecting livestock [8,9,10], and more generally, STL are known for their antitumor, antimicrobial, antioxidant, hepatoprotective, antiprotozoal, and antiaging properties [11]. Both CGA and STL were also studied for their anti-inflammatory effect in vitro as well as in vivo [6,12]. Some other molecules present in chicory roots and leaves, such as flavonoids or tannins, were studied for their specific health effects and their contents correlated with antioxidant [13] and anti-nematode [14] properties.

In this work, we looked at three classes of molecules that are part of the composition of the chicory flour: fructose, CGA and STL. We experimented on the murine model and performed a nutrigenomic analysis, a metabolic hormone assay and a metagenomic analysis of the gut microbiota, to target the major health effects and molecular mechanisms of action in these compounds. Results were supported by in vitro apoptosis detection on human hepatocellular carcinoma HepG2 cells, using flow cytometry, by in vitro inflammatory cytokines secretion assay on promonocytic human cell line U937, differentiated into macrophages, and by a cell-free system antioxidant assay.

## 2. Materials and Methods

Chicory product obtaining and chemical analysis of their composition

Roots from industrial chicory (*Cichorium intybus* var. *sativum*) provided by Florimond-Desprez Veuve et Fils SAS (Cappelle-en-Pévèle, France) were processed by Leroux SAS (Orchies, France) and the corresponding flour was delivered by Waast Mill (Mons-en-Pévèle, France). This flour is a trade product that comes from a mixture of several genotypes with a significantly different chemical composition. An aqueous decoction of this flour was produced to feed the mice as described by Pouille et al. [2]. A mixture of water/methanol (1:1) was added to either 100 mg of a randomized sample of chicory flour or 100 mg of dry residue obtained after lyophilisation of chicory decoction. The samples were mixed for 10 min at 80 °C, using a ThermoMixer^®^ (Eppendorf AG, Hamburg, Germany) at 2000 rpm, followed by 10 min of sonication at 80 °C using an ultrasonic bath at 35 kHz. The samples were centrifuged at 4 °C for 10 min at 12,000 rpm. The supernatant was diluted 20 times with methanol/water (50/50). All samples were filtered through 0.22 μm PTFE membrane filters before analysis by ultra-performance liquid chromatography coupled to high-resolution mass spectrometry (UPLC-HRMS) and nuclear magnetic resonance (NMR). UPLC-HRMS analysis was performed on an ACQUITY UPLC I-class chain coupled with the Vion IMS Q-TOF high resolution mass spectrometer, equipped with an electrospray (ESI) (Waters, Manchester, UK) ionization source (Z-spray) and an additional spray for the reference compound (Lock Spray). A double detection in the positive and negative mode was performed by ESI mass spectrometry (range 50–2000 Da) and by a PDA diode array detector (UV detection between 190–500 nm). Separation was performed using a KINETEX Biphenyl (100 × 2.1 mm, 1.7 μm) column (Phenomenex) heated at 55 °C with a mobile phase (solvent A (0.1% formic acid in water) and solvent B (0.1% formic acid in methanol)) flow (0.55 mL·min^−1^) and the same gradient elution as our previous study [15]. The spectra obtained were acquired and processed with UNIFI software (version 1.9.4, Waters) and enabled us to generate the data matrix for untargeted metabolomics analyses with classical parameters. Calibration mixture solution of target metabolites at concentrations of 0.5, 1, 1.5, 2, 2.5, 3, 3.5, 4, 4.5 and 5 μM were prepared by dilution in three replicates. Area values of the extracted ion chromatograms were transferred to Excel (Microsoft Excel 2011 v. 14.7.2, Microsoft, Redmond, WA, USA).

The NMR untargeted metabolomics protocol was adapted from our previous study [16]. Briefly, the supernatant (500 μL) was dried under vacuum and then dissolved in 800 μL of deuterated solvent prepared in a mixture of (1:1) Methanol-d4: KH_2_PO_4_ buffer (0.1 M) in D_2_O at pH 6.0 with TMSP (0.0125%), NaN_3_ (0.6 mg·mL^−1^), and maleic acid (1 mM). Then, the samples were briefly vortexed, sonicated, and centrifuged. The supernatant was placed in 5-mm NMR tubes and then used for NMR analysis. All NMR spectra were acquired at 300 K with a Bruker Avance III 600 MHz spectrometer operating at 600.13 MHz for ^1^H, and 150.91 MHz for ^13^C, using a 5-mm double resonance broadband probe, equipped with z-gradient (BBFO 5 mm tube). For quantitative analysis, classical 1D ^1^H-NMR spectra were collected using 128 scans of 131 K data points and a spectral width of 8417 Hz with a relaxation delay of 25 s. For metabolomics profiling, a NOESY-1D water suppression pulse sequence was used and generated spectra were collected using 256 scans of 131 K data points and a spectral width of 8417 Hz with a relaxation delay of 25 s. NMRProcFlow web application [17] was used to generate the untargeted metabolomics data matrix and the quantitative ^1^H-NMR data. Bins with the lowest overlapping signals were kept (0.9 to 3.25 ppm and 4.5 to 8.5 ppm). Signals of maleic acid were used to calculate the absolute concentration of targeted metabolites. A combined data matrix with NMR (161 bins) and UPLC-HRMS (positive (77 ions) and negative (71 ions) data) untargeted analyses were generated. The percentage of the relative standard deviation (% RSD) was calculated for all metabolic features in each condition and the features with % RSD greater than 25% were removed due to variability. Metabolite pick areas were expressed in percentage (100% corresponds to the mean for chicory flour). Heatmap data was clustered (Ward’s method was used to form hierarchical clustering) and visualized (using the pheatmap-package, version 1.0.12).

Animal experiments and ethical statements

Male BALB/cOlaHsd 8-week-old mice were used for experiments in agreement with Directive 2010/63/EEC for the protection of animals used for scientists and in accordance with Law 2012-10 (2012) and 2013-118 (2013). The protocol was approved by the Ethics Committee in charge of animal experiments. The mice were randomly divided into six groups (*n* = 5/group) and housed in a controlled environment (with a temperature of 22 °C, a 12 h/12 h light/dark cycle and ad libitum access to standardized food and water). Mice were fed with an aqueous decoction of root flour (Chic) and with three other molecules or classes of molecules: fructose (Fru), CGA and STL water solution as indicated in Table 1. The mice gavages consisted in a daily force-feeding of 500 µL of Chic, Fru, CGA or STL besides the standard chow (Diet A04C-10, Scientific Animal Food and Engineering, Augy, France). For the chicory flour, the decoction corresponded to 30 mg root powder/mouse/day that was considered close to human equivalent weight/body mass for a moderated alimentary dose [18]. The administered dose of Fru, CGA and STL was calculated to correspond to the concentration of these compounds in the aqueous extract of chicory flour for daily consumption. This estimation was performed taking into account the variability of these compounds in 5 different chicory genotypes [19] as indicated in Table 1. Controls underwent an equivalent force-feeding with water (Ctr1), the solvent used for chicory decoction and fructose solution, and with 0.83% DMSO (Ctr2) as CGA and STL solutions were prepared with this diluent [20,21]. Six groups of mice were used (Ctr1, Ctr2, Chic, Fru, CGA and STL), nourished for 30 days and individual body weight was regularly registered (File S1). At the end of this period, mice for each condition were sacrificed, the central core of the liver left lobe was cut into cubes, a short segment (1 cm) of the colon was also cut and enterocytes were harvested from upper ileal segments by mucosa scraping method. These tissues were immediately frozen in liquid nitrogen and stored as individual samples at −80 °C for transcriptomics. The feces were individually harvested before the treatment (D0) and after 30 days (D30), and were stored at −80 °C for microbiota analyses. For hormonal assays, 100 µL of blood from the caudal vein was sampled from each animal 30 min after gavage at D30. The blood from each mouse was collected in tubes containing 20 µL 10% EDTA anticoagulant solution and 1 µL dipeptidyl peptidase-4 inhibitor (DPP4-010, Marck, Milipore, Darmstadt, Germany), then centrifuged at 14,000 rpm for 10 min, the plasma containing supernatants collected and stored at −80 °C.

### 2.1. RNA Extraction and Microarray Analysis

Total RNAs were extracted from hepatic and colon tissues using RNAspin columns (Macherey-Nagel, Düren, Germany). For ileum cells, the NucleoZOL (Macherey-Nagel) kit was used. RNA quality was checked with Nanodrop and absorbance ratios A260/280 and A260/230 were found between 2.0 and 2.2. RNA quality was also examined by RNA ScreenTape Analysis (Agilent) and a minimal RNA integrity number (RIN) of 0.8 was required for all samples.

For the microarray analysis, groups of mice (*n* = 3) were used for each treatment: (1) a group that received a decoction of chicory flour for 30 days as previously described, (2) a group that received a solution of fructose (Fru) for 30 days, (3) a group that received a solution of CGA for 30 days, (4) a group that received a solution of STL for 30 days, (5) two control groups with standard drinking water (Ctr1 and Ctr2) as mentioned before. Three tissues were analyzed: liver, ileum enterocytes and caecum.

Agilent Whole Mouse Genome Microarray Sure Print GE 4 × 44 v2 with oligonucleotide 45,220 probes was used to study the gene expression profile. RNA amplification, staining, hybridization and washing were conducted according to the manufacturer’s specifications. Slides were scanned at 5 µm/pixel resolution using the GenePix 4000B scanner (Molecular Devices Corporation, Sunnyvale, CA, USA). Images were used for grid alignment and expression data digitization with GenePix Pro 6.0 software. Expression data were normalized by Quantile algorithm. The 3 control samples were filtered for *p* value < 0.05 and the average was calculated for each gene. A fold change (FC) value was calculated between individual treated samples and the mean of corresponding controls. Differentially expressed genes (DEGs) were selected for a threshold >2.0 or <0.5. Functional annotation of DEGs was based on NCBI GenBank and related genes’ physiological processes were assigned with NCBI, AmiGO 2 Gene Ontology and UniProt. KEGG pathway analysis was also used to identify relevant biological pathways of selected genes. All microarray data have been submitted to the NCBI GEO archive for functional genomics data with the accession number GSE190056.

### 2.2. In Vivo Hormone Detection

Plasma concentrations of leptin and GIP (glucose-dependent insulinotropic polypeptide) were assessed using antibody-immobilized beads specific to each hormone in a Milliplex^®^ Map Kit (Millipore Corporation, Billerica, MA, USA) according to the manufacturer’s instructions. Kit sensitivity was 19 pg·mL^−1^ for leptin and 1 pg·mL^−1^ for GIP. The quantification was carried out using the Luminex^®^ 100/200 (Luminex Corporation, Austin, TX, USA) system and the Luminex xPONENT^®^ for LX100/200 software.

### 2.3. Microbiota Analysis

Total DNA content from mice feces was extracted according to NucleoSpin DNA Stool Kit (Macherey-Nagel, Düren, Germany). The sequencing was done by the GIGA genoproteomic platform of Liège University (Belgium). For sequencing the amplification of the V1-V3 region of the 16S rDNA and the library preparation were performed with the following primers: direct (5′-GAGAGTTTGATYMTGGCTCAG-3′) and inverse (5′-GAGAGTTTGGCTCAG-3′). Each PCR product was purified with the Agencourt AMPure XP Ball Kit (Beckman Coulter, Pasadena, CA, USA), and subjected to a second round of PCR for indexing, using Nextera XT index 1 and 2 primers. After purification, the PCR products were quantitated using the Quant-IT PicoGreen (Thermo-Fisher Scientific, Waltham, MA, USA) and diluted to 10 ng·μL^−1^. A final qPCR quantification of each library sample was performed using the KAPA SYBR FAST qPCR Kit (KapaBiosystems, Wilmington, NC, USA) before standardization, pooling, and sequencing on a MiSeq sequencer using v3 reagents (ILLUMINA, San Diego, CA, USA).

Data processing was performed using, respectively, the MOTHUR v1.44 package and the VSearch algorithm [22] for alignment, clustering and chimer detection as previously described by Gérard et al. [23]. After cleaning process, sequences were clustered into operational taxonomic units (OTUs) at 97% of identity. Alignment and taxonomical identification were performed with MOTHUR using SILVA v1.32 database of full-length 16S rDNA gene sequences. A rarefied table of 10,000 reads by sample was used for further analysis. Reads were finally aggregated into phylotypes at the phylum and genus taxonomic level.

All analyses were performed by comparing experimental groups to their respective controls. Normality was controlled with the Shapiro–Wilk test and homogeneity of variances with Bartlett’s test. Statistical paired differences were assessed by ANOVA and Tukey’s test.

PRISM 7 (GraphPad Prism 6.0, Windows Inc., San Diego, CA, USA) was used and differences were considered significant for a *p* value < 0.05. All the biosample raw reads have been deposited at the National Center for Biotechnology Information (NCBI) and are available under BioProject accession number PRJNA799887. Data obtained from NGS analysis were analyzed for the alpha diversity with Shannon index and graphical representations were performed using GraphPad Prism version 8.00 for windows, GraphPad Software, and beta-diversity with the principal component analysis (PCoA) using the FactoMineR package in R version 3.5.2 (r-project.org).

### 2.4. In Vitro Cytotoxicity Studies

The cytotoxic effect of samples was analyzed to determine the maximum concentrations that could be used for the other tests. The cytotoxic effect was performed using the cell counting assay-8 (CCK-8) (CK04, Tebu-Bio). The kit was used according to the manufacturer’s instructions. HepG2 and U937 cells were seeded in 96-well culture plates at 8 × 10^4^ cells·cm^−2^ and 3 × 10^5^ cells·cm^−2^ respectively. After reaching confluency, the growing medium was replaced by samples diluted at increasing concentrations in Dulbecco’s modified Eagle’s medium (DMEM, 4.5 g·L^−1^ glucose) or in Roswell Park Memorial Institute medium (RPMI-1640). Culture medium was used as a negative control of cytotoxicity. The cells were incubated at 37 °C for 24 h and the cytotoxic effect of samples was immediately determined.

### 2.5. In Vitro Apoptosis Assay

The human hepatocellular carcinoma HepG2 cells were grown at 37 °C, 5% CO_2_ atmosphere, in DMEM supplemented with 10% fetal bovine serum (FBS), 100 U·mL^−1^ penicillin, 100 μg·mL^−1^ streptomycin, and 2 mM glutamine. After reaching 80–90% confluency, HepG2 cells were seeded into 24-well culture plates at 8 × 10^4^ cells·cm^−2^ six days before experiment. The culture medium was discarded and cells were washed with PBS. Apoptosis was induced by adding different amounts of lyophilized chicory decoction or D-fructose powder (0.2%, 0.5%, 1%, 2% and 3%, *m*/*v*), and different concentrations of CQA or STL (5, 10, 20, 50, 75 and 100 µM). Resveratrol at a concentration of 200 µM [24] was used as a positive control and DMEM medium as a negative one. The cells were then incubated at 37 °C for 24 h. Early and late apoptotic events were analyzed by flow cytometry (Invitrogen™ Attune™ NxT Flow Cytometer) with the Alexa Fluor 488 annexin V/Dead Cell Apoptosis Kit (Invitrogen, Waltham, MA, USA) according to the manufacturer’s kit manual.

### 2.6. In Vitro Inflammatory Cytokines Secretion Assay

The promonocytic human cell line U937 was cultured in RPMI 1640 medium supplemented with 10% FBS, 100 U·mL^−1^ penicillin, 100 μg·mL^−1^ streptomycin, and 2 mM glutamine in a humidified 5% CO_2_ atmosphere at 37 °C. For macrophage differentiation, U937 cells were seeded at approximately 3 × 10^5^ cells·cm^−2^ in 24-well plates with 60 ng·mL^−1^ phorbol-12-myristate-13-acetate (PMA) for 48 h. Adherent cells were washed with PBS before a 2 h incubation with LPS (10 µg·mL^−1^; LPS from *E. coli* O26:B6, Millipore Corporation) and samples. Cell supernatants were collected on ice and centrifuged (1500 rpm 5 min) to eliminate cell debris. The supernatants were aliquoted and stored at −80 °C until further analysis. Secreted Il-1β and TNF-α were quantified using Millipore Human High Sensitivity T Cell kit (EMD Millipore, Darmstadt, Germany) according to the manufacturer’s instructions. The quantification was carried out using the Luminex^®^ 100/200 (Luminex Corporation, Austin, TX, USA) system and the Luminex xPONENT^®^ for LX100/200 software. The cytokine IL-8 was quantified using a human IL-8/CXCL8 Quantikine^®^ ELISA kit (R&D Systems, Inc., Minneapolis, MN, USA). Medium samples were diluted 100-fold according to the kit recommendations.

### 2.7. Cell-Free Systems Evaluating Antioxidative Sample Effects

All reagents were purchased from Sigma (Saint-Quentin Fallavier, France). The concentration of the starting samples to be diluted was standardized at 10 mg of dry matter per mL. After all assays, the maximal percentage of inhibition obtained for each reactive oxygen species (ROS) at the highest concentration for each sample was also calculated and half-maximal inhibitory concentration (IC_50_) was determined when practicable.

The superoxide anion inhibition was estimated in a cell-free model adapted from that previously described by Aruoma et al. [25]. Briefly, O_2_^−^ was produced by the xanthine (0.1 mM)/xanthine oxidase (50 mU·mL^−1^) system in a Hank’s HEPES buffer (HH) (pH 7.42) and then incubated at 25 °C for 15 min with increasing sample concentrations ranging from 0 to 100 µL·mL^−1^ of reaction mixture (equivalent to 0 to 1 mg of dry matter per final mL) and with 0.017 mM equine ferricytochrome c (FerC). The appearance of pink coloration corresponds to the FerC reduction (into ferrocytochome C) by the remaining superoxide anions non-inhibited by the samples. An inhibition control cuvette also contained 300 µM cysteine. Spectrophotometry method was used to measure the absorbance at the wavelength of 550 nm. Then, O_2_^−^ concentrations were calculated thanks the Beer–Lambert law using the ferrocytochrome C extinction coefficient (21.1 × 10^3^ L·mol^−1^·cm^−1^). All measurements were performed against a blank cuvette, containing all reagents and samples except xanthine oxidase to avoid any interference. Results were converted into nmol·mL^−1^ and expressed in bar diagrams ± SD according to the sample volumes involved per mL of reaction mixture. The hydroxyl radical inhibition by the control solutions or sample studied was assayed using a method adapted from that described by Halliwell, Gutteridge and Aruoma [26]. HO^.^ was produced in each tube in 20 mM KH_2_PO_4_ buffer at pH 7.4 with 10^−11^ nmol of hydrogen peroxide per mL. For that, Fenton’s reaction was initiated by adding EDTA-Fe^2+^ (100 µM FeCl_3_, 104 µM ethylene diamine tetraacetic acid and 100 µM ascorbic acid). The solutions/sample concentrations (the same as above described for H_2_O_2_) were added for HO^.^ to be then inhibited at least partially. Deoxyribose (3 mM) was added to be degraded by the remaining hydroxyl radicals. After boiling for 20 min, malondialdehyde (MDA) was generated as a result of 14 mM thiobarbituric acid and 147 mM trichloroacetic acid. The resulting pink chromogen was measured by spectrophotometry (532 nm). HO^.^ inhibition control tubes were also made with 300 µM cysteine. HO^.^ was expressed in nmol·mL^−1^, using a standard curve obtained from increasing H_2_O_2_ concentrations. The data was presented as bar diagrams ± SD according to the sample volumes involved per mL of reaction mixture. Statistical analysis was conducted using 6 independent assays (GraphPad Prism 7 software for Windows, Inc., San Diego, CA, USA). Provided the population was normal (Shapiro–Wilk’s test at the 5% level) as well as variance homogeneity obtained (non-significative Bartlett’s test at the 5% level), ANOVA was performed (overall Fisher’s test, *p* < 0.05, followed by the ad-hoc Tukey’s test, *p* < 0.05). When ANOVA was not applicable, the non-parametric test of Kruskal–Wallis (*p* < 0.05) followed by Dunn’s test (*p* < 0.05) were performed.

## 3. Results

Whole chicory flour (Chic) and three compounds of chicory roots (Fru, CGA and STL) were separately analyzed for their health effects in mice. A whole transcriptomic analysis was conducted using Agilent DNA microarrays to investigate their nutrigenomic effects, the plasmatic hormonal levels were assayed using Luminex technology, and a fecal microbiota metabarcoding analysis was realized by Illumina 16S rDNA sequencing. We carried out parallel in vitro analyses to investigate apoptotic, anti-inflammatory and antioxidant effects of these different compounds.

### 3.1. Composition of Chicory Products

An untargeted metabolomic approach was conducted using UPLC-HRMS and ^1^H-NMR data, obtained from the analysis of two types of preparation i.e., chicory flour and its decoction. Similar metabolite profiles were observed when comparing both preparations. However, metabolomic fingerprinting revealed that the extraction of the metabolites during the preparation of the decoction from the chicory flour was slightly less effective. We estimated that during the decoction preparation, several metabolites were extracted in proportion to 70–80% compared to their content in the flour (considered to be 100%) (File S2). A possible explanation may be that in the decoction, the extraction of metabolites in hot water could be incomplete, in particular for hydrophobic compounds; the stirring could also play an important role in the observed results. Metabolites that were used in this work for animal feed were subsequently targeted by quantitative ^1^H-NMR and also by UPLC-HRMS and results are presented in Table 2. The content of CGA and STL in the decoction seems, generally, similar to that of flour. We found that only lactucin content was higher in the decoction, while the content of fructose was slightly lower in the latter. Despite these minor quantitative differences, the quality composition of the chicory decoction remains identical to that of the chicory flour and, therefore, its use for a mouse diet could be relevant for evaluating the chicory roots effects.

### 3.2. Nutrigenomic Analyses

**Figure 1:**
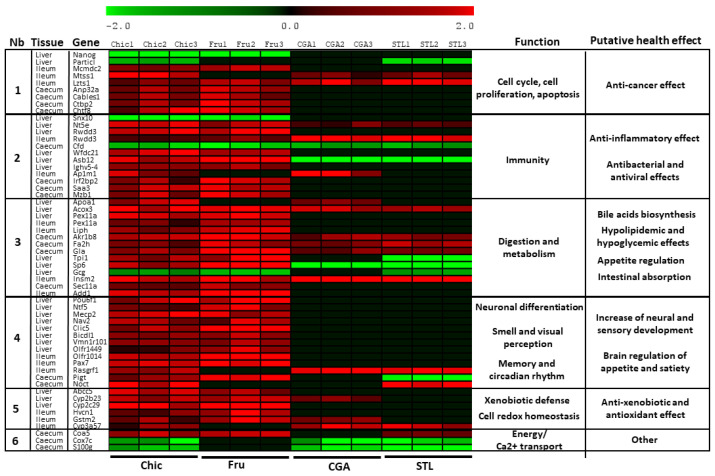
Gene expression profiles in hepatic tissue, ileum cells and caecum of mice after different diets. Log2 fold change of gene expression was represented individually for mice (1–3) and gene-related physiological processes and putative health effect are represented on the right part of the graph. Chic—chicory flour diet; Fru—fructose diet; CGA—chlorogenic acids treatment; STL—sesquiterpene lactones treatment.

Diet-induced gene expression profiles were analyzed in the liver, ileum enterocytes and caecum of mice after 30 days of daily ingestion of aqueous extracts of Chic, Fru, CGA or STL solutions. A total of 57 profiles were found differentially expressed during the chicory diet in these different tissues (Figure 1, File S3). A first group of nine DEGs, modified by the chicory diet, is involved in cell proliferation and apoptosis, and their deregulation suggests a cell growth arrest and a putative anti-cancer effect. A second group of 12 DEGs is involved in immune response, and this gene deregulation suggests a putative anti-inflammatory effect and a strong response to bacteria and viruses. An important group of 14 DEGs (group 3) is involved in digestion and metabolism, pointing to bile acid biosynthesis, hypolipidemic and hypoglycemic effects, appetite regulation and intestinal absorption increase. In the 4th group, we noticed a stimulation of the neural and sensory development, suggested by the up-regulation of 13 genes involved in neuron differentiation and development, memory, smell and visual perception, and also in circadian rhythm regulation. The anti-xenobiotic and putative antioxidant effects were indicated by six DEGs (group 5), and finally, we found three genes involved in energy metabolism and calcium transport that were also deregulated, but their putative health effect could be less discernible (group 6). When following these profiles in the other diet conditions (Fru, CGA and STL), we observed that in the vast majority of cases, the fructose offers similar deregulation as the chicory (48 DEGs with similar profile), reflecting the importance of fructose for the functional effect of the chicory flour. CGA and STL are also involved in these deregulations, as CGA induces a similar profile as the chicory flour for 18 genes and STL also for 18 genes. We noticed only four opposite profiles between chicory flour and CGA or STL diets, probably due to the administration of these compounds apart from the food matrix. We observed that the same functions appear to be deregulated in the three analyzed tissues, even if different genes respond specifically in each of them; only two genes (Rwdd3 and Pex11a) were found similarly expressed in the liver and ileum and were separately recorded.

### 3.3. In Vivo Hormonal Assays

**Figure 2:**
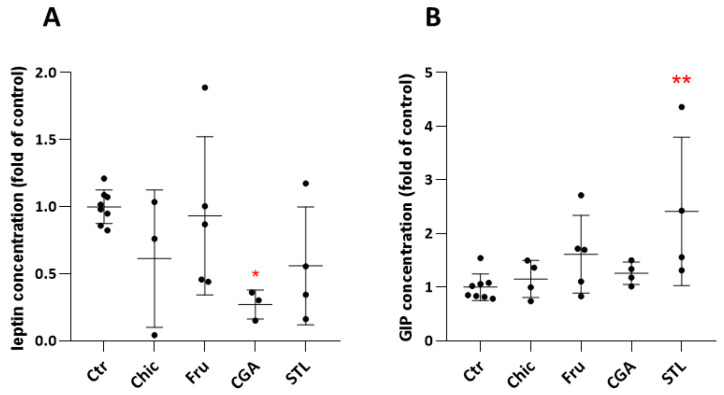
Leptin (**A**) and GIP (**B**) level in mice plasma after 30 days of chicory, fructose, CGA and STL supplemented diet. Control values were pooled together (Ctr). Plasmatic hormone levels were expressed as a ratio of the control level. Statistical analysis was performed using one-way ANOVA and Dunnett’s multiple comparisons test (* *p* < 0.05; ** *p* < 0.01 against control).

Slight differences were observed in plasmatic hormone levels following chicory flour intake. These differences were not significant in the Chic condition, but other compounds have proven to be significantly involved in these modifications. Thus, CGA decreased circulating leptin level and STL increased GIP level (Figure 2, File S4). The decrease in leptin level could be associated with a hypolipidemic effect, and GIP increase, with a hypoglycemic effect. These effects were equated with the mice body weight evolution and standard food consumption during the diets (File S1).

### 3.4. Metagenetic Analysis of Mice Microbiota

**Figure 3:**
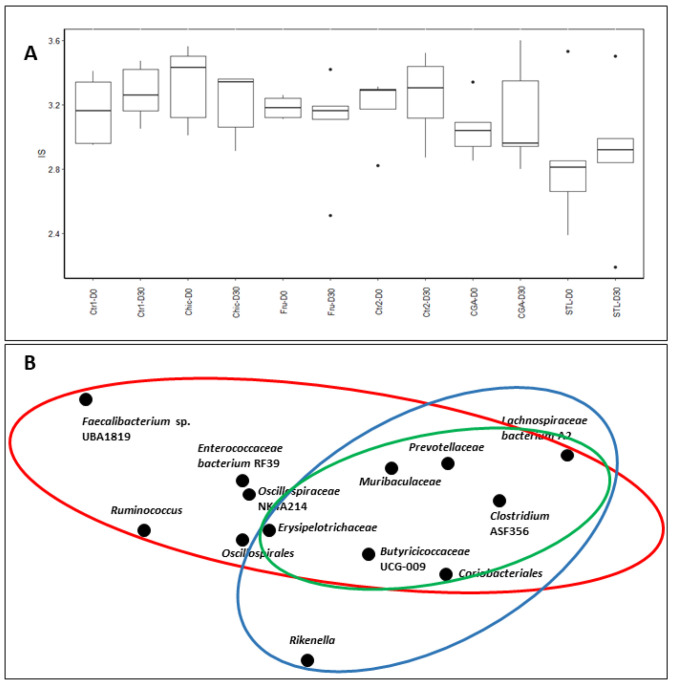
Microbial diversity in fecal microbiota of mice after chicory (Chic), fructose (Fru), chlorogenic acids (CGA) or sesquiterpene lactones (STL) supplemented diet. (**A**) Alpha diversity was illustrated by the Shannon index (IS) that indicates a stabilized diversity for all subject groups (*p* > 0.05). (**B**) Principal Coordinates Analysis (PCoA) plots (beta-diversity) of affected taxa during different diets. Relative abundance obtained from sequencing the 16s rRNA gene in fecal samples was represented for taxa providing differences during chicory flour diet. Red circle mainly delimits the fructose effect, blue circle delimits the STL effect and green circle the CGA effect.

16S-rRNA gene-targeted metagenomic analysis was performed in the individual fecal samples of mice (*n* = 5) for each diet and the bacterial richness and phylogenetic composition of the microbiota were then estimated. After sequence processing, a total of 68 different operational taxonomic units (OTUs) were identified with an abundance > 0.1% and the average number of OTUs per individual was 703 ± 60 (File S5). The alpha diversity Shannon index indicated a stabilized diversity to the average of 2.8–3.5 for all sample groups, and no significant modifications were found during the Chic, Fru, CGA or STL diets (Figure 3A). Beta diversity among different conditions, as a comparison of taxa abundance, was considered by Principal Coordinate Analysis, which revealed a common but also specific effect of the chicory flour compounds (Figure 3B). Fructose impacted a larger number of taxa but CGA and STL also share an important role in these changes and present several particular effects.

**Figure 4:**
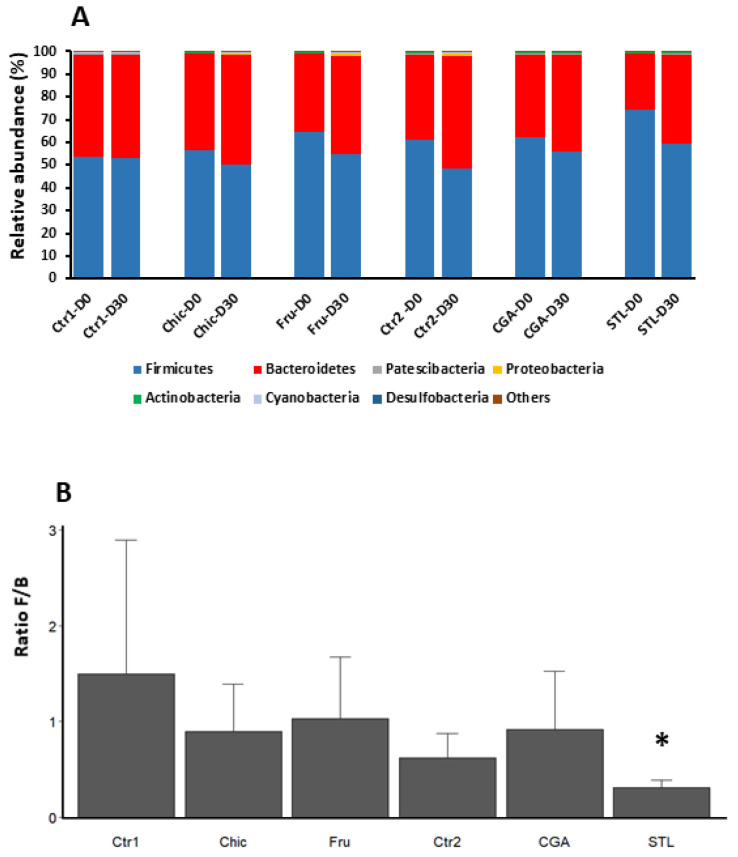
Changes in bacterial phyla abundance. (**A**) Relative abundance (%) of phyla in mice microbiota before (D0) and after 30 days (D30) of chicory (Chic), fructose (Fru), chlorogenic acids (CGA) or sesquiterpene lactones (STL) supplemented diet. Relative abundances detected by NGS are expressed as means. Phyla with abundance under 0.1% are grouped in “Others”. (**B**) Standardized abundance ratio relative to D0 of *Firmicutes* and *Bacteroidetes* (Tukey’s test, *n* = 5/group, * for *p* < 0.1). Chic—chicory flour; Fru–fructose; CGA—chlorogenic acids; STL—sesquiterpene lactones; Ctr1—control related to Chic and Fru diet; Ctr2—control related to CGA and STL diet.

**Figure 5:**
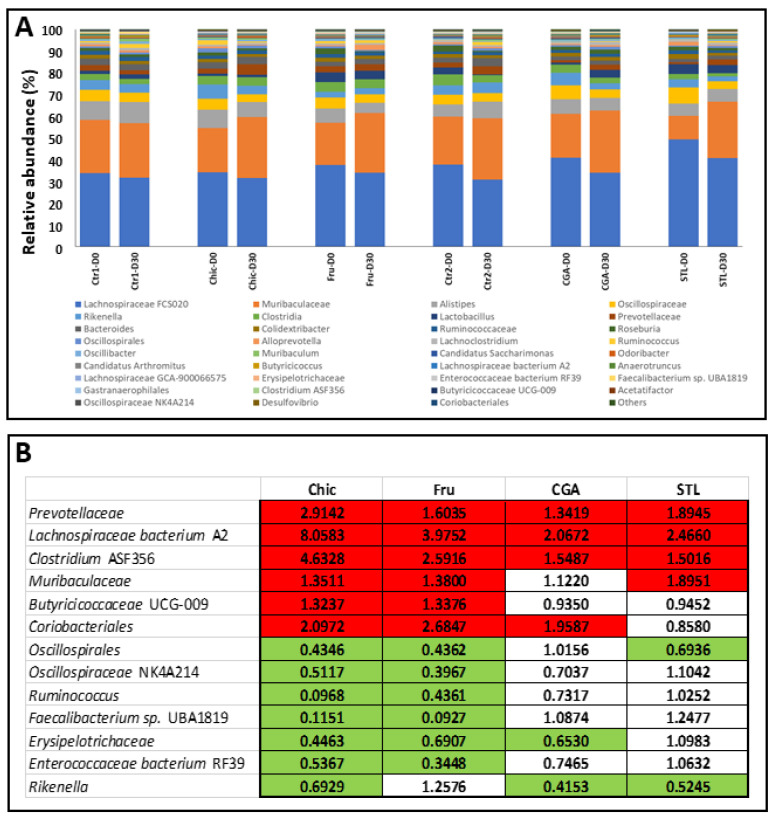
Relative abundance of main genera in mice fecal microbiota after chicory flour (Chic), fructose (Fru), chlorogenic acids (CGA) or sesquiterpene lactones (STL) diet. (**A**) Relative abundances of genera (%) are indicated when their values are >0.1%. Genera with a low relative abundance were assigned as “Others”. (**B**) Heatmap representing the fold change of standardized abundance ratio. Increased abundance compared to control was considered when FC > 1.30 (red squares) and decreased when <0.70 (green squares).

The bacterial composition representing the phylum and genus relative abundance for major OTU is shown in Figure 4 and Figure 5, respectively. The most abundant phyla across all young subjects were *Firmicutes*, *Bacteroidetes*, *Patescibacteria*, *Proteobacteria*, *Actinobacteria*, *Cyanobacteria* and *Desulfobacteria* (Figure 4A). The most present were *Firmicutes* and *Bacteroidetes* and the ratio *Firmicutes*/*Bacteroidetes* (F/B) was found to be slightly diminished after 30 days of the chicory diet, probably due to the STL activity, as the only STL diet was found to induce a significant decrease (Figure 4B and File S6).

At the genus level, all changes observed in the relative abundance after 30 days of different treatments are represented in Figure 5. Quantitative modifications were observed in the abundance of several taxa after the different diets (Figure 5A). These modifications were closely followed as fold change of standardized abundance ratio (condition vs. control) (Figure 5B). Of the taxa, 13 were filtered for their relative abundance (>0.1%) consistently, in all samples, and presented modifications during the different diets. Six of them increased in abundance during the chicory diet, and seven of them decreased. The three analyzed compounds of the chicory flour (Fru, CGA and STL) triggered a similar increase in the relative abundance as the chicory flour for three taxa (*Prevotellaceae*, *Lachnospiraceae bacterium* A2 and *Clostridium* ASF356). Fructose alone impacted 5 taxa (*Butyricicoccaceae* UCG-009, *Oscillospiraceae* NK4A214, *Ruminococcus, Faecalibacterium* sp. UBA1819, and *Enterococcaceae bacterium* RF39), while CGA affected only the *Erysipelotrichaceae*. The results showed that, sometimes, two different classes of molecules could be involved together in changes in the abundance, as for *Muribaculaceae*, *Coriobacteriales*, *Oscillospirales* and *Rikenella*. A basic part of these results was validated by qPCR (File S7).

### 3.5. In Vitro Evaluation of the Apoptotic Effect

**Figure 6:**
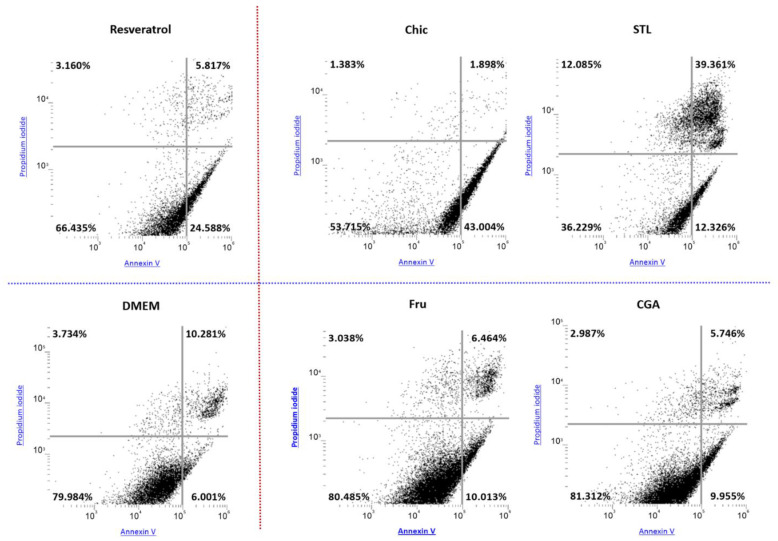
Dot plots illustrating the apoptotic effect of chicory flour and its compounds on HepG2 cells. HepG2 cells were treated for 24 h with chicory decoction (Chic), fructose (Fru), CGA and STL solutions at different concentrations and analyzed using propidium iodide and Annexin V. Representations were selected among the most elevated concentration of each compound (3% for Chic and Fru; 100 µM for CGA and STL). Resveratrol at a concentration of 200 µM was used as a positive control, and DMEM as a negative one. The top left quadrant of each plot represents unviable cells, the top right quadrant represents necrotic cells or cells in late apoptosis. The bottom left quadrant represents viable cells and the bottom right quadrant represents early apoptotic cells. Total apoptotic cells were calculated by adding the top right and bottom right quadrant’s content. Samples illustrated in the top of the figure (Resveratrol, Chic and STL) provided an increased apoptotic effect compared to the other samples.

**Figure 7:**
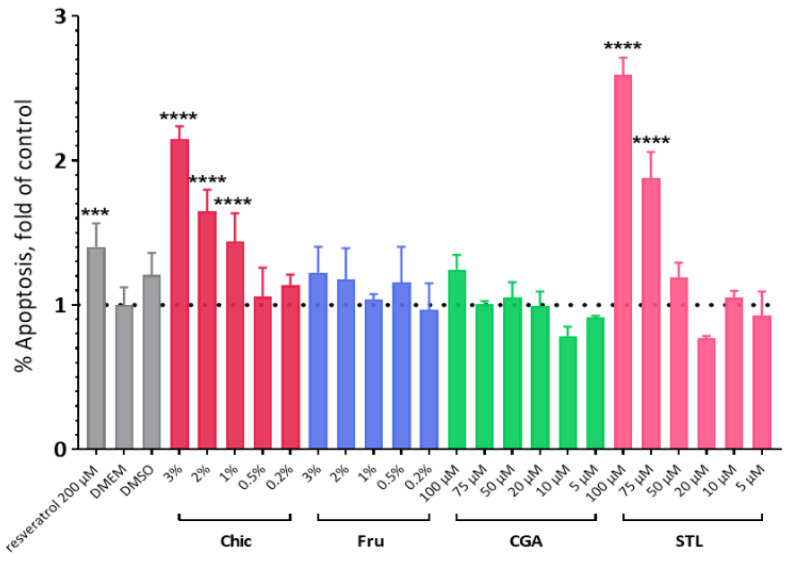
Apoptosis induction on HepG2 cells. HepG2 cells were treated with various concentrations of a chicory flour decoction (Chic) or D-fructose solution (Fru) (from 0.2% to 3%), and also with chlorogenic acids (CGA) or sesquiterpene lactones (STL) (from 5 µM to 100 µM) for 24 h. Resveratrol at a concentration of 200 µM was used as a positive control and DMEM and DMSO as negative ones. As no significant variations were registered between negative controls, the apoptosis induction in each condition was compared to the DMEM control and expressed as a ratio. Statistical analysis was performed using one-way ANOVA and Dunnett’s multiple comparisons test (**** *p* < 0.0001; *** *p* < 0.0005 compared with the DMEM control).

The effect of raw chicory flour decoction on apoptosis was studied by labelling HepG2 cells with Annexin V and propidium iodide and analyzed by flow cytometry. The amount of total apoptotic cells was calculated by considering both cells in early and late apoptosis (Figure 6); each value was reported to their respective control (DMEM). The chicory decoction induced a concentration-dependent increase in the number of apoptotic cells. Fru, CGA and STL were also tested separately and results showed that only STL at 75 µM and 100 µM induced a significant apoptotic effect (Figure 7).

### 3.6. In Vitro Anti-Inflammatory Effect

**Figure 8:**
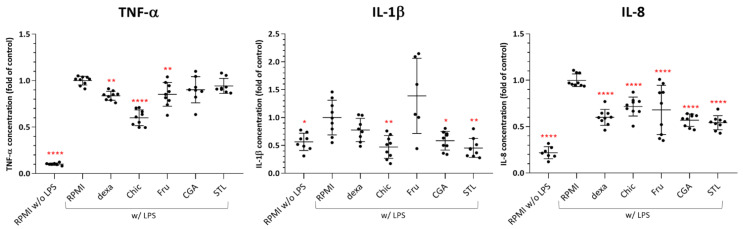
Effects of chicory and its compounds on TNF-α, IL-1β and IL-8 production by human U937 macrophages. U937 were differentiated with PMA before being inflamed with LPS (w/LPS) and put in contact with samples for 2 h. Dexamethasone (dexa) was used as a negative control of inflammation. Efficiency of LPS inflammation was controlled (RPMI w/o LPS) in each test. Cytokine levels were expressed as a ratio of the control (RPMI w/LPS) level. Statistical analysis was performed using one-way ANOVA and Dunnett’s multiple comparisons test (* *p* < 0.05; ** *p* < 0.005; **** *p* < 0.0001 against control). dexa: 20 µM of dexamethasone; chic: 1% of chicory flour decoction; Fru: 1% of D-fructose; CGA: 20 µM of chlorogenic acids mix; STL: 20 µM of sesquiterpene lactone mix.

Chicory and its compounds at the highest non-cytotoxic concentration (data not shown) were screened for their anti-inflammatory activity in vitro using U937 cells, differentiated in macrophages and their capacity to secrete cytokines. Three pro-inflammatory cytokines (TNF-α, IL-8, IL-1β) were quantified in the cell media. As a control, stimulation with LPS significantly increased the secretion of TNF-α, and interleukin IL-8, (*p* < 0.0001) and IL-1β (*p* < 0.05) (Figure 8). The addition of the chicory decoction markedly reduced the secretion of all cytokines (*p* < 0.005) in inflamed cells. The decrease in interleukin levels seems to be due to CGA and STL effect and the reduction of TNF-α can probably be linked to the fructose effect.

### 3.7. In Vitro Antioxidant Effect

**Figure 9:**
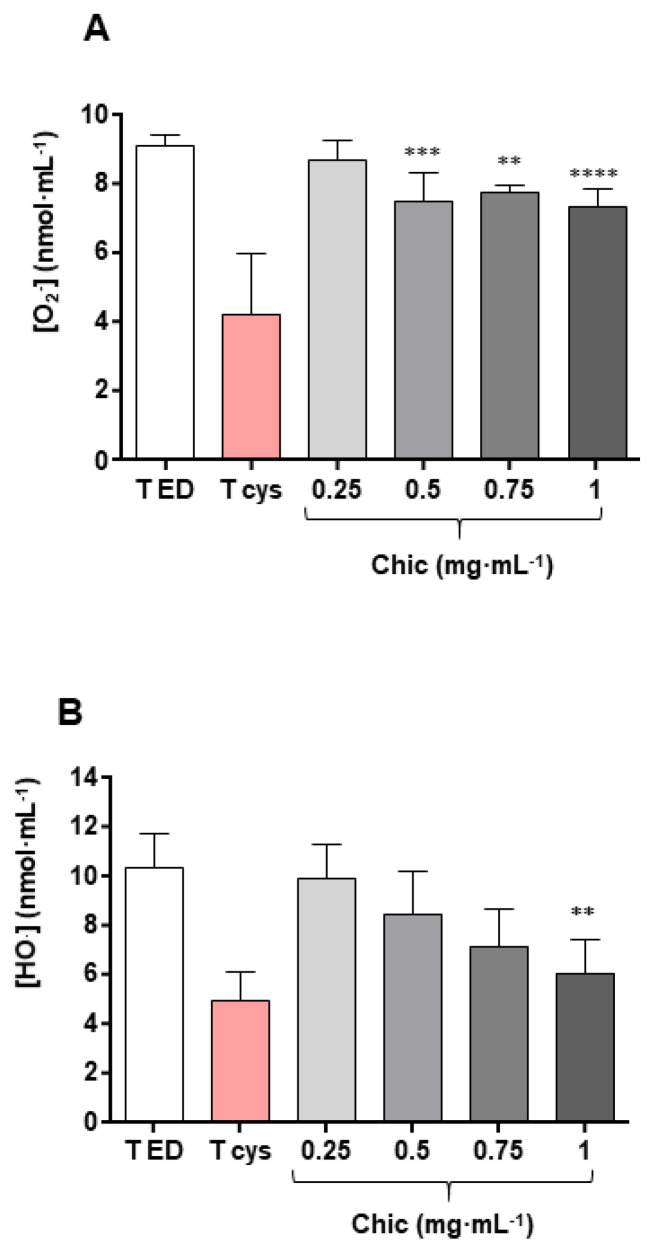
Antioxidative effects of chicory flour. Superoxide anion inhibition (**A**) and hydroxyl radical inhibition (**B**) of chicory flour decoction were observed by contrast with the effect of the negative control (T ED) containing distilled water, and the positive control containing cysteine (T cys). The sample decoction of chicory flour (Chic) was tested at increasing concentrations, ranging from 0.25 to 1 mg of dry matter equivalent per final mL. Statistical analysis for *n* = 6 independent assays were performed with ANOVA: overall Fisher’s test, *p* < 0.0001; Tukey’s test, ** *p* < 0.01, *** *p* < 0.001, **** *p* < 0.0001 (**A**) and Kruskal-Wallis test, *p* = 0.0017; Dunn’s test, ** *p* < 0.01 (**B**).

The antioxidant effect of the chicory flour was checked in a cell-free model for the superoxide anion and hydroxyl radical inhibition. The aqueous extracts of chicory flour triggered a significant decrease in superoxide anions, beginning from 0.5 mg·mL^−1^, and also a decrease in hydroxyl radicals that became significant, beginning from 1 mg·mL^−1^ (Figure 9).

## 4. Discussion

Chicory flour is a food product used in pastry and, at the same time, a functional food with multiple health benefits [2]. Several molecules or classes of molecules that enter the composition of chicory flour are possible effectors in these effects. This work was carried out to decompose the effects of chicory flour and to better understand the role of three of its components, Fru, CGA and STL. To do this, in vivo murine experimental analyses were carried out using an aqueous decoction of chicory, whose composition was found similar and quantitatively close (up to 70–80%) to that of flour. Analyses were performed to intercept nutrigenomic, hormonal and metagenomic changes that were subsequently correlated with events observed in vitro on human cells or in acellular systems.

### 4.1. The Anti-Cancer Effect of the Chicory Roots as Observed in an In Vivo Murine and In Vitro Human Cell Models

A chicory supplemented diet triggered the deregulation of nine genes involved in cell proliferation and apoptosis (Figure 1). In addition, this diet induced an apoptotic effect in HepG2 cells in vitro (Figure 6 and Figure 7). To determine which compound from chicory is responsible for this effect, we looked at the role of Fru, CGA and STL on gene expression and on HepG2 cells phenotype.

In liver tissue, we found two down-regulated genes under chicory treatment. The first one, *Nanog,* is a transcriptional factor that helps embryonic stem cells to maintain pluripotency by suppressing cell determination factors [27]. As it is highly expressed in cancer stem cells, this gene functions as an oncogene to promote carcinogenesis [28,29], being described as a prognostic and predictive cancer biomarker [30]. Its down-regulation in Chic and Fru dietary conditions suggests that chicory flour, via its fructose content, can lead to the arrest of cells engaged in cancer proliferation. The second gene in this group, *Particl,* is involved in the response to irradiation and affords an RNA binding platform for genomic silencers, such as DNA methyltransferase 1 and histone tri-methyltransferases, to reign in the expression of tumor suppressors [31]. This gene was correlated to the expression of tumor suppressor genes, as it operates an active feedback silencing mechanism upon the putative tumor suppressor mat2a, to limit its expression [32]. The knockdown of this gene resulted in inverse changes in *Wwox* transcripts levels, which is also known as coding for a tumor suppressor [33]. As we found the *Particl* gene down-regulated by the Chic and STL diets, we can suggest that chicory flour, via its STL content, can lead to an increase in tumor suppression, probably by apoptosis [34]. Our results on HepG2 cells indicated that only STL induced a significant apoptotic effect that could be related to *Particl* gene down-regulation.

In ileum cells, we found three up-regulated genes under the Chic diet. *Mcmdc2* is an essential gene for meiotic recombination-mediated repair [35] and fructose seemed to be the compound responsible for its up-regulation. *Mtss1* plays an important role in metastasis blocking, by governing the metastatic nature of cancer cells [36,37], and its up-regulation was led by the CGA and STL diets. *Lzts1* codes for a tumor suppressor that may stabilize the active CDC2-cyclin B1 complex and thereby contributes to the regulation of the cell cycle and the prevention of uncontrolled cell proliferation [38]. All chicory compounds (Fru, CGA and STL) seemed to participate in the up-regulation of this gene.

In caecum tissue, four genes were up-regulated during the chicory diet. *Anp32a* codes for a protein that has been found to be decreased or absent in malignant tumors, and to modulate cell growth by regulating p38 and AKT activity [39]. *Cables1* codes for a vital cell cycle regulator, dysregulation of which has been associated with a large number of human malignancies [40]. *Ctbp2* codes for a negative regulator of cell proliferation, and *Chtf8* is also involved in the cell cycle and, equally, in telomere maintenance via semi-conservative replication [41]. These genes were found to be up-regulated by the chicory diet, and fructose seems to be the bioactive component for all of them.

We observed that fructose plays a major role in the deregulation of genes involved in cell proliferation regulation, but CGA and STL can also impact several key genes, especially those involved in metastasis suppression. STL alone seems to be responsible for one important apoptosis-related gene expression, and this observation could be associated with in vitro results (Figure 8), showing a significant apoptotic effect of STL on HepG2 cells. Our observations are consistent with other studies showing the effect of chicory extracts on the regulation of cell proliferation and apoptosis in different human cancer cell lines [42,43].

### 4.2. The Anti-Inflammatory Effect as Observed in an In Vivo Murine and In Vitro Human Cell Models

All three analyzed tissues responded by an increase in the anti-inflammatory effect during the chicory diet. The liver responded by the deregulation of *Snx10*, *Nt5e* and *Rwdd3*, ileum, also by the *Rwdd3* deregulation and the caecum by the *Cfd* gene deregulation (Figure 1). SNX10 presents a crucial role in macrophage polarization and inflammation, and the loss of SNX10 function was proposed to be a potentially promising therapeutic strategy for inflammatory bowel disease [44]. The down-regulation of this gene suggests an anti-inflammatory effect, triggered by the chicory flour, and among its components by the fructose only. NT5E is a marker of lymphocyte differentiation, and this enzyme contributes to the anti-inflammatory properties of afferent lymphatic endothelial cells in humans and mice [45]. Fructose, but also CGA and STL, could induce an up-regulation of the gene coding for this enzyme. RWDD3 is involved in the negative regulation of NF-κB transcription factor activity [46] and its up-regulation by the chicory diet could be attributed to the fructose in the liver. In ileum cells, in addition to fructose, CGA and STL also increased the expression of this gene. CFD is a component of the alternative complement pathway [47], involved in the inflammatory response. Its down-regulation during the chicory diet could be attributed to all analyzed compounds: fructose, CGA and STL.

An anti-inflammatory response from chicory roots has already been observed by Ripoll et al. [48] and Matos et al. [49], who investigated the immunomodulatory mechanisms of STL across the modulation of inflammatory pathways in vivo, on rat and mouse models and, respectively, in vitro, on human intestinal mucosa Caco-2 cells. Our observations in vivo were performed over nutrigenomic analyses, under chicory flour, fructose, CGA and STL supplementation (Figure 1) and indicated a deregulation of four genes involved in anti-inflammatory responses. Thereafter, our in vitro observations (Figure 8) showed that chicory supplementation in mice led to a decrease in TNF-α, IL-1β, IL-8 levels. The fructose, CGA and STL were pointed as effector molecules. CGA and STL seemed to be affecting IL-1β and IL-8 levels more, and fructose was responsible for TNF-α reduction.

### 4.3. Antibacterial and Antiviral Effect as Observed in an In Vivo Murine Model

During the chicory diet, seven genes involved in the antimicrobial effect and innate immune system were impacted (Figure 1). Thus, *Wfdc21*, *Asb12*, *Ighv5-4*, *Ap1m1*, *Irf2bp2*, *Saa3* and *Mzb1* were found up-regulated under the Chic diet, except *Ap1m1,* which was impacted by the CGA, while all the others were up-regulated by the fructose intake. The antibacterial effect of a CGA-rich extract on several common pathogens was already suggested by in vitro experiment [7] and the honey containing fructose was also described as a potential anti-bacterial agent [50].

### 4.4. Hypolipidemic and Hypoglycemic Effects, Appetite Regulation and Intestinal Absorption as Observed in an In Vivo Murine Model

Chicory has been described as a digestive remedy for both humans [51,52] and animals [53,54,55]. Hypolipidemic and hypoglycemic effects were already observed in mice [2]. We support these data with new in vivo nutrigenomic and hormonal effects in the present study. Liver tissue, ileum enterocytes and caecum tissue responded to the chicory flour diet by the up-regulation of seven genes, resulting in lipid metabolism modifications with, consequently, hypolipidemic effects (*Apoa1*, *Acox3*, *Pex11a*, *Akr1b8*, *Fa2h* and *Gla*), the deregulation of five genes, triggering a hypoglycemic effect and affecting the appetite regulation (*Tpi1*, *Sp6*, *Gcg*, *Insm2*, *Sec11a*), and also a gene playing an important role in intestinal absorption (*Add1*) (Figure 1).

Gene coding for APOA1, which is the major component of the high-density lipoprotein complex, playing a key role in the metabolism of triglycerides [56] and clear fats, including cholesterol [57], was found up-regulated under the CGA diet. Gene coding for ACOX3 is essential for bile acid formation [58], with a role in lipid destructuration, and was up-regulated by all chicory compounds (Fru, CGA and STL). Gene coding for LIPH, which is also involved in the triglyceride catabolism [59], and for PEX11A that is known to have a major impact on lipid metabolism [60,61], were found deregulated in both liver and ileum, being impacted by the fructose only. AKR1B8, that regulates fatty acid synthesis [62], FA2H, that is involved in galactosphingolipid synthesis [63], and GLA, that is involved in sphingolipids metabolism [64], were also impacted by all bioactive effectors: fructose, CGA and STL (Figure 1). All these deregulations could trigger a hypolipidemic effect, and this consequence is also suggested by in vivo Luminex assay (Figure 2). The circulating level of leptin was reduced after 30 days of chicory supplementation and significantly altered by the CGA diet. Being directly linked to the body fat distribution [65], a decrease in plasmatic leptin could be associated to the hypolipidemic effect. In our study, body weight was not significantly altered (Supplementary File S1), however, a slight decrease in body weight was observed for Chic fed mice.

Concerning glycemic effect, we observed that gene coding for TPI1, which is involved in glycolysis [66], and for SP6, involved in hepatic gluconeogenesis [67], were up-regulated during the chicory diet. The responsible bioactive compound seems to be the fructose that induced the same deregulation as the chicory flour when administrated alone. We know that honey containing fructose exerts a hypoglycemic effect [68], and so, obviously, the fructose contained in the chicory could act similarly.

The appetite regulation was already described as an important effect in chicory root consumption [2,18]. We found *Gcg* gene coding for pro-glucagon to be down-regulated. This gene, which plays a key role in glucose metabolism and homeostasis [69], was impacted by the fructose and STL. The gene coding for INSM2 that stimulates the pancreatic endocrine cell differentiation and, thus, the insulin biosynthesis [70], was up-regulated by all chicory compounds. We also found that gene coding for Sec11a, involved in incretin synthesis [71], was up-regulated under the chicory diet and that fructose seems to be the bioactive compound that impacts this gene. More than that, the circulating GIP level was increased after chicory consumption and significantly higher with STL supplementation (Figure 2). It is known that GIP is a primary incretin hormone, secreted from the intestine, to stimulate insulin secretion from pancreatic β cells and, thus, plays an important role in glucose metabolism [72].

Finally, the intestinal absorption was already observed to be impacted by a chicory supplementation [73], but the mechanism was unidentified. We found that gene coding for ADD1, which is involved in actin cytoskeleton organization [74], and consequently in various intestinal functions as cell motility, endothelial adhesion junctions, absorption, etc. [75], was up-regulated under chicory flour and, respectively, fructose bioactive action.

### 4.5. Neural and Sensory Development as Observed in an In Vivo Murine Model

This study highlighted the effect of chicory on the neural and sensory development by nutrigenomic analyses. A group of 13 genes was up-regulated under chicory intake (Figure 1). Among these genes, we note that the one coding for POU6F1 is involved in brain development and neural plasticity [76], NTF5 related to formation and maintenance of neuronal connections [77] and to taste development [78], MECP2 is essential for the normal function of nerve cells [79], and BICDL1 is a key component of vesicular transport in developing neurons [80]. Further, in this group, we found the gene coding for NAV2 that is involved in the development of different sensory organs [81], CLIC5, which is required for normal hearing [82], VMN1R101, OLFR1449 and OLFR1014, with a role in smell perception [83,84]. Another worth noting is the deregulation of gene coding for PAX7, which plays a role in neural crest development [85], RASGRF1, known for its role in long-term memory [86] and PIGT, described as implied in neuron differentiation [87]. Surprisingly, we also found a significant up-regulation in gene coding for NOCT, which is involved in the circadian rhythm regulation [88]. Other than *Rasgrf1,* which was up-regulated under CGA, and *Noct,* which was up-regulated under CGA bioactive impact, the rest of the genes were deregulated under fructose impact. Excess fructose consumption has been related to metabolic syndrome and obesity, and more generally, to the feeding behavior and a hunger-like state in the brain [89]. In chicory plants, the fructose is present in a low quantity in roots (3–6 mg/g dry matter depending on genotype). Its regular consumption, enclosed in the food matrix, was assimilated to a benefit as appetite booster [90] or appetite regulator [18] and the mechanism of its action could most probably interfere with genes involved in neural and sensory development.

### 4.6. Anti-Xenobiotic and Antioxidant Effect as Observed in Both In Vivo Murine and In Vitro Cell-Free Models

The chicory decoction triggered an up-regulation of genes involved in anti-xenobiotic and antioxidant effects, such as *Abcc5,* which codes for a transmembrane pump essential for the elimination of certain toxins [91], *Hvcn1* and *Gstm2,* which are involved in the maintenance of redox homeostasis [92,93], and also several genes coding for cytochrome P450 polypeptides, such as CYP2B23, CYP2C29 and CYP3A57, that are known to be involved in xenobiotic metabolism [94]. Fructose was found to be the bioactive compound involved in this effect for four genes, but also CGA (for three genes) and STL (for one gene) (Figure 1). We also observed an important effect of the chicory decoction on the hydroxyl radical and on the superoxide anion (Figure 9). All these observations are in agreement with other studies and confirm the antioxidant effect of the chicory, most likely due to fructose, CGA and STL [6,11,95].

### 4.7. Microbiota Modifications

Fecal specimens were used for a 16S RNA targeted metagenomics analysis to estimate gut microbiota changes during the different diets. The alpha diversity indicates a relative homogeneity of responses, and the beta diversity indicates that the different chicory compounds trigger common, but also specific, modifications in bacterial taxa abundance (Figure 3). The ratio between the *Firmicutes* and *Bacteroidetes* phyla was calculated for different treatments, and a decrease in this ratio was registered for the Chic diet, probably due to the STL effect as the STL diet triggered a significant decrease in the F/B ratio (Figure 4). A change of F/B ratio may be important because this was described to be correlated with digestion and metabolic processes. In humans, this ratio was found to be significantly increased in patients developing type 1 diabetes and also obesity [96,97]. In mice, this ratio was already observed to be impacted by the chicory diet [2,18], which is also in accordance with our observations. In addition to this, we were able to detect that STL is the class of molecules that mostly modifies this ratio.

Daily feeding with the chicory flour decoction triggered changes in the relative abundance of several bacterial genera (Figure 5). Several taxa increased in abundance (*Prevotellaceae*, *Lachnospiraceae bacterium* A2, *Clostridium* ASF356, *Muribaculaceae*, *Butyricicoccaceae* UCG-009, *Coriobacteriales*), while others decreased (*Oscillospirales*, *Oscillospiraceae* NK4A214, *Ruminococcus*, *Faecalibacterium* sp. UBA1819, *Erysipelotrichaceae*, *Enterococcaceae bacterium* RF39, *Rikenella*) and these variations were tracked in Fru, CGA and STL diets to understand the role of these compounds in the different modifications.

Thus, the abundance of *Prevotellaceae* was found to be increased in the Chic diet and similarly impacted by all tested compounds. This gut bacterial taxon was found in humans, correlated with Parkinson’s Disease [98,99], its decrease indicating a disease state. *Prevotellaceae* was assigned as a psychobiotic [100], in other words, a group of beneficial bacteria that influences bacteria–brain relationships and exerts anxiolytic and antidepressant effects, characterized by changes in emotional, cognitive, systemic, and neural indices [101]. Short chain fatty acid (SCFA) producers were also recognized as psychobiotics [100], and we noticed in our study at least four bacterial taxa, known as butyrate, and other SCFA producers that were impacted by chicory flour and their abundance were found increased during fructose but also by the CGA and/or STL: *Lachnospiraceae bacterium* A2, *Clostridium* ASF356, *Muribaculaceae*, *Butyricicoccaceae* UCG-009 [102,103]. These observations go near the neural and sensory development, suggested by the nutrigenomic analysis, and suggest an improvement of neuronal functions during a chicory supplemented diet.

*Coriobacteriaceae* seemed to be positively affected under chicory uptake conditions and mainly influenced by fructose and CGA. These bacteria carry out functions of importance, such as the conversion of bile salts and steroids, as well as the activation of dietary polyphenols [104] and its modifications could be associated to the hypolipidemic effect of the chicory that was also observed across the nutrigenomic analysis. Our study showed that *Oscillospirales*, *Oscillospiraceae* NK4A214 and *Ruminococcus* taxa were negatively impacted by the chicory and fructose diets. These taxa were described as being negatively associated with high fatty liver index (FLI) and connected with the non-alcoholic fatty liver disease (NAFLD) [105,106,107], and their decrease in our study supports even more the hypolipidemic and hepatoprotector effect of the chicory, and especially of the fructose.

*Faecalibacterium* sp. UBA1819, a succinate-producing bacterium that was described to be involved in the increase in sleep fragmentation and blood pressure [108], was found with a diminished abundance during the chicory diet, probably due to the fructose content. This observation suggests a hypotensive effect of the chicory uptake.

The chicory diet across fructose, but also CGA and STL, triggered the decrease in the abundance of several taxa, known as mainly pathogenic or inflammatory factors, such as *Erysipelotrichaceae*, *Enterococcaceae bacterium* RF39 and *Rikenella* [109,110].

We can conclude for the microbiota analysis that together, these modifications in taxa composition surprisingly merged with many nutrigenomic impacts of the chicory uptake: effects on the neural development (by the psychobiotics as *Prevotellaceae* or different SCFAs producers increased abundance), hypolipidemic effects (by the *Coriobacteriaceae* increased abundance, and *Oscillospirales*, *Oscillospiraceae* NK4A214 and *Ruminococcus* decreased abundance), anti-inflammatory and anti-pathogenic effects (suggested by the *Erysipelotrichaceae*, *Enterococcaceae bacterium* RF39 and *Rikenella* decreased abundance). A hypotensive effect was also suggested by the *Faecalibacterium* sp. UBA1819 decreased abundance. As for the transcriptomic analyses, the fructose was found to play an important role in bacterial richness, being involved in the abundance modification of 12 bacterial taxa. The CGA was also observed to exert a noticeable influence for six taxa, and the STL for the other six taxa.

## 5. Conclusions

Chicory as a functional food has been studied in the form of root, transformed into flour, for pastry or bakery use, or more generally in the food industry. To identify the main effector molecules of the chicory, we split the composition of this flour into several classes of molecules. Inulin, the main component of the chicory root, has already been the subject of studies, showing its exclusively prebiotic effect [2]. In this work, fructose, CGA and STL were tested in daily administration in mice, and a set of analyses were performed. The gene expression profile of the experimental animals, following the different diets, indicated several deregulated biological functions, leading to several putative health effects: anti-cancer, anti-inflammatory, antimicrobial effect, and metabolic effect concerning bile acid biosynthesis, lipid and carbohydrate metabolism, appetite regulation and intestinal absorption. A regulatory effect has also been observed in many genes affecting neuronal and sensory development, suggesting a regulation of energy homeostasis. In addition, anti-xenobiotic and antioxidant effects were also observed. All these effects seem similar in the three different analyzed tissues, even though different genes appear to govern these responses in each tissue.

These effects were then recognized by complementary in vivo analyses, such as hormonal assay, and also by in vitro analyses on cultured cells or on acellular assays, to highlight the apoptotic, anti-inflammatory and antioxidant effects. A 16S RNA targeted metagenomic analysis completed the panel of health effects of the chicory flour and its components, and showed the role of fructose, CGA and STL in the development of several SCFA producers, with a psychobiotic role, bacteria with hypolipidemic, hepatoprotective and also a hypotensive role.

**Figure 10:**
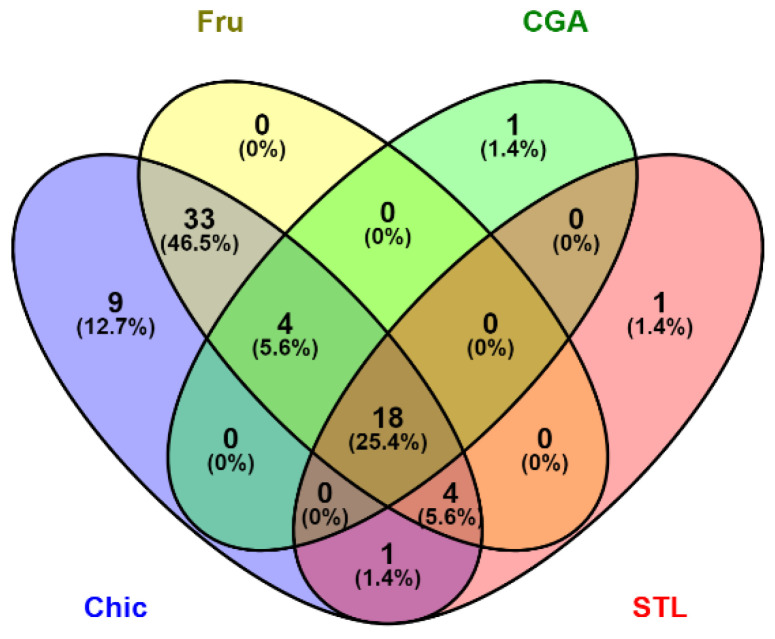
Venn diagram illustrating an estimation of the most important effects of the chicory and its tested compounds: fructose (Fru), chlorogenic acids (CGA) and sesquiterpene lactones (STL). Number of DEGs was considered in transcriptome analysis, and number of modified taxa in metagenetic analysis. Results of hormone assay were constricted to significant modification as well as for in vitro analyses as significant response was considered 1 and a non-significant one was considered 0. A score was calculated by totaling these events for each diet. The Venn diagram’s platform (http://bioinfogp.cnb.csic.es/tools/venny/index.html accessed on 21 January 2022) was used to cross the data.

Hence, our study explicitly targeted these effects on gene expression, hormone-releasing and microbiota modifications and pointed out the role of different effector molecules contained in chicory roots: fructose, CGA and STL. All these responses are summarized in Figure 10 and File S8. Fructose seems to be the most involved in these activities, contributing to approximately 83% of all recorded responses. Almost half of the fructose effects were observed at the metabolism level and brain appetite regulation. CGA and STL have shown a specific role for all different effects, with an estimated 23 and 24% contribution, respectively. Further studies are certainly needed to test the role of other classes of molecules, as well as the defined role of the food matrix. Chicory genotype breeding and/or CRISPR-Cas9 tool [111] should be able to guide us even more quickly in the complete deciphering of different effects and effectors of chicory flour on health.

## Figures and Tables

**Table 1:** Composition of Fru, CGA and STL solutions used for feeding mice.

Treatment	Fru	CGA		STL			
Compound	Fructose	3-CGA	3,5-CGA	DHLc	Lc	DHLp	Lp
µg/day of each compound	166.35	2.52	3.44	1.83	3.88	0.29	2.22
µg/day of total mix	166.35	5.90		8.20			

3-CGA: 3-mono-*O*-caffeoylquinic acid; 3,5-CGA: 3,5-di-*O*-caffeoylquinic acid; DHLc: 11β,13-Dihydrolactucin; Lc: Lactucin; DHLp: 11β,13-Dihydrolactucopicrin; Lp: Lactucopicrin. The administered doses are based on results obtained for the content of chicory roots and reported to 30 mg/day of chicory flour.

**Table 2:** Major metabolites analysed by quantitative ^1^H-NMR and UPLC/ESI-HRMS in chicory root flour and decoction.

Compounds (mg/g Dry Matter)	Flour		Decoction	
	Mean	SD	Mean	SD
Fructose ^a^	3.43	0.12	2.60	0.26
3-CGA ^b^	0.55	0.02	0.66	0.01
3,5-diCGA ^b^	0.43	0.01	0.43	0.01
Lc ^c^	0.05	0.01	0.11	0.01
Lp ^c^	0.23	0.02	0.21	0.01
DHLc ^c^	0.14	0.01	0.17	0.01
DHLp ^c^	0.05	0.01	0.03	0.01

^a^ quantitative ^1^H-NMR, bin (3.54) corresponding to ^1^H linked to C1, ^b^ UPLC/ESI-HRMS in negative ionization mode and ^c^ in positive ionization mode. 3-CGA: 3-mono-*O*-caffeoylquinic acid, *m*/*z* 353.0878 at RT 1.08 min; 3,5-diCGA: 3,5-di-*O*-caffeoylquinic acid, *m*/*z* 515.1195 at RT 4.27 min; Lc: Lactucin, *m*/*z* 299.0889 at RT 3.02 min; Lp: Lactucopicrin, *m*/*z* 433.1257 at RT 5.77 min; DHLc: 11β,13-Dihydrolactucin, *m*/*z* 279.1227 at RT 2.59 min; DHLp: 11β,13-Dihydrolactucopicrin, *m*/*z* 435.1414 at RT 5.72 min.

## Data Availability

Transcriptomic and metagenomics data are archived in GEO database and NCBI’s Sequence Archive and will be publicly accessible at the same time as the publication of the article.

## Supplementary Materials

The following supporting information can be downloaded at: https://www.mdpi.com/article/10.3390/nu14050957/s1, File S1. Mice body weight evolution (A) and standard food consumption (B) during chicory (Chic), fructose (Fru), chlorogenic acids (CGA) and sesquiterpene lactones (STL) supplemented diet for 30 days. File S2. Heatmap of metabolite signals. File S3. Gene expression profiles in mice liver tissue, ileum cells and caecum of mice during different diets. File S4. GIP and leptin level in mice plasma, after 30 days of chicory, fructose, CGA and STL diet. File S5. The operational taxonomic units (OTUs) identified with an abundance >0.1% in fecal microbiota of mice after different diets. Diets were supplemented with chicory flour (Chic), fructose (Fru), chlorogenic acids (CGA) and sesquiterpene lactones. File S6. Standardized abundance ratio (relative to D0) for Firmicutes and Bacteroidetes phyla after 30 days of diet in mice. File S7. Microbial profiling using quantitative PCR. File S8. Estimation of the most important effects of the chicory and its tested compounds: fructose (Fru), chlorogenic acids (CGA) and sesquiterpene lactones (STL).
