# Supplementary material for: Chicory: Understanding the Effects and Effectors of This Functional Food
Source: Nutrients. 2022 Feb 23;14(5):957. doi: 10.3390/nu14050957 (PMC8912540; doi:10.3390/nu14050957)
Supplement: Supplementary file 1 [file nutrients-14-00957-s001.zip › File S1.pdf]

File S1. Mice body weight evolution (A) and standard food consumption (B) during chicory (Chic), fructose (Fru), chlorogenic acids (CGA) and sesquiterpene lactones (STL) supplemented diet for 30 days.

Body weight (grams) was continuously assessed during diet (n = 5 animals per group D0-D30). Data are expressed as means.

A

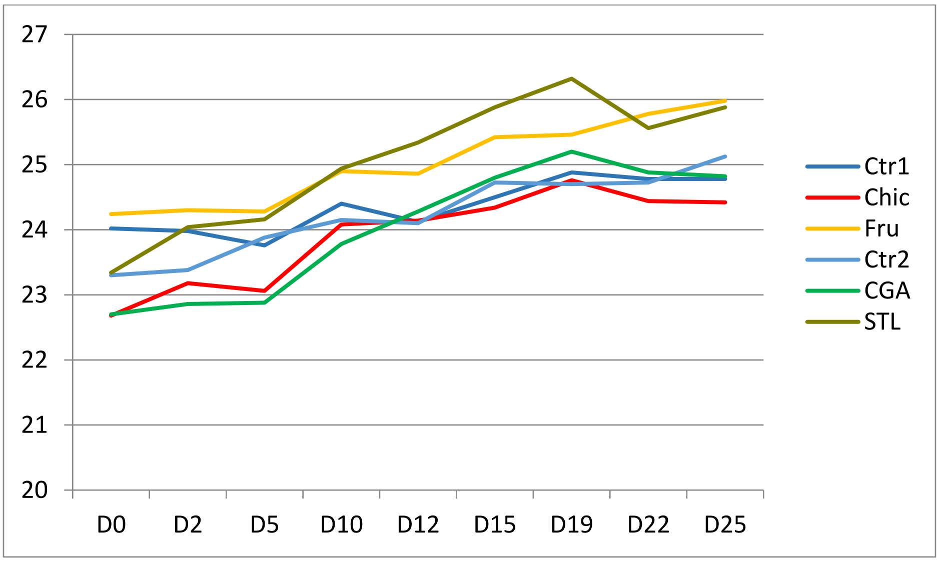

(A) Diet with chicory flour seems to trigger a no significant but sustained decrease of body weight in mice after 10 days of forced feeding. Fructose and STL administrated alone as supplement of diet generate a slight increase of weight in mice after 5-10 days of forced feeding.

B

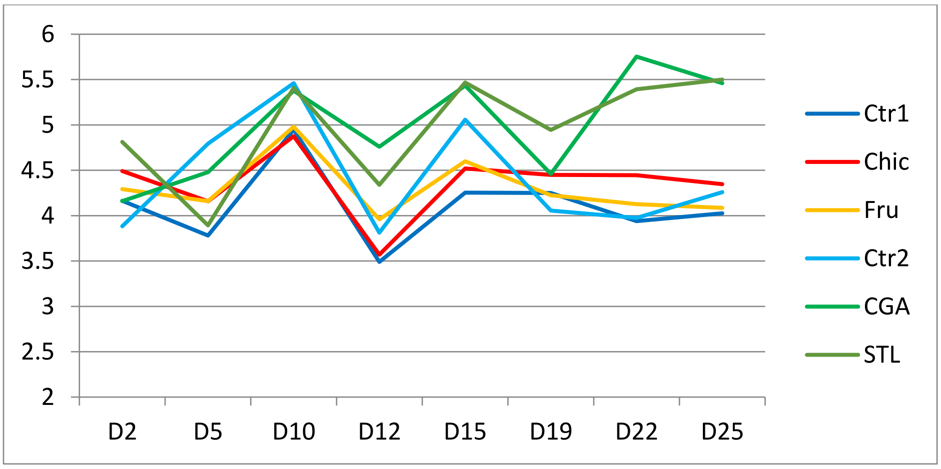

(B) When CGA and STL were administrated alone, mice increased progressively their food consumption. There was no increase observed for chicory or fructose supplemented diets.
