# Supplementary material for: Chicory: Understanding the Effects and Effectors of This Functional Food
Source: Nutrients. 2022 Feb 23;14(5):957. doi: 10.3390/nu14050957 (PMC8912540; doi:10.3390/nu14050957)
Supplement: Supplementary file 1 [file nutrients-14-00957-s001.zip › File S4.pdf]

File S4. GIP and leptin level in mice plasma, after 30 days of chicory, fructose, CGA and STL diet.

Statistical analysis was carried out using One-way ANOVA and Sidak's multiple comparisons test. Means ± SD were calculated (n ≥ 3) for each treatment. Mean values of all controls were standardized at 1 value.

		Control	Chic	Fru	CGA	STL
**Leptin**	n	8	3	5	3	4
	mean ± SD	1 ± 0.1259	0.6133 ± 0.5126	0.9323 ± 0.59	0.2716 ± 0.1083	0.5588 ± 0.4403
	p-val	X	0.4378	0.9953	0.0414	0.2415
**GIP**	n	8	4	5	4	4
	mean ± SD	1 ± 0,2492	1,15 ± 0,3461	1,614 ± 0,7258	1,26 ± 0,2095	2,416 ± 1,383
	p-val	X	0.9903	0.3544	0.9324	0.0086
