# Supplementary material for: Chicory: Understanding the Effects and Effectors of This Functional Food
Source: Nutrients. 2022 Feb 23;14(5):957. doi: 10.3390/nu14050957 (PMC8912540; doi:10.3390/nu14050957)
Supplement: Supplementary file 1 [file nutrients-14-00957-s001.zip › File S7.pdf]

File S7. Microbial profiling using quantitative PCR

In order to validate results obtained by Illumina sequencing, the abundance of 2 phyla (*Firmicutes* and *Bacteroidetes*) and 4 genera (*Prevotellaceae*, *Clostridium ASF356*, *Oscillospira*, and *Ruminococcus*) were checked by qPCR method.

Total bacterial DNA was extracted from ~40 mg of fecal sample using according to NucleoSpin DNA Stool Kit (Macherey-Nagel, Germany). The relative amount of bacterial phyla of interest was checked by measuring DNA abundance of the 16S rRNA gene sequences of each group of bacteria using the AriaMx Real-Times PCR system (Agilent, USA) and group-specific primers following previously described protocols (Parnell and Reimer, 2012; Chaplin et al., 2016). The group-specific primers are shown in Table S7_1. Primers were verified with Multalin software (Corpet, 1988), and Primer3 (Untergasser et al., 2012) and finally in silico checked with the Probe Match tool in RDP (Ribosomal Database Project) (Cole et al., 2014). Primer specificity, amplification efficiency and the limit of detection were determined using serial dilutions of a standard DNA.

Quantitative PCR was performed in AriaMx Real-Time PCR System (Agilent), on 96-well microplates. Amplifications were carried out in a final volume of 15μL containing 0.75 μl of each primer (10 μM) (Sigma-Aldrich, USA), 7.5 μL iQ SYBR Green Supermix (Biorad) and either 6 μL of DNA or water (no-template control). The thermal cycling conditions were as follows: an initial denaturation step at 95°C for 3 min followed by 40 cycles of denaturation at 95°C for 15s, primer annealing at 60°C for 30s, extension at 72°C for 30s and a fluorescence acquisition step at 72°C for 10 min. DNAs extracted from fecal samples were amplified in triplicate for each primer set and the mean value was used for statistical analysis.

For each pair of primers, a melting curve was made to verify the specificity of the amplification. The efficiency and threshold cycle (Ct) were calculated with the Agilent.AriaMx software. Total Bacteria is a broad-range primer that identifies the conserved region of the 16S rRNA encoding gene for a wide range of bacteria. Data obtained by this qPCR reaction gives the amount of total bacteria present in the sample. Then the relative quantification of each target bacteria or group was normalized with the total bacteria content in the sample, following the mathematical model defined by Livak K., & Schmittgen T. (2001). Data were calculated as $2^{-\Delta\Delta Ct}$ means and comparisons were performed using one-way ANOVA with Tukey's and Student's tests (* $p < 0.1$, ** $p < 0.05$).

Table S7_1. Primer sequences for qPCR

Specificity	Primer	Sequence (5' – 3')	Amplicon length (bp)	Tm (°C)	Reference
**Total bacteria**	Forward	ACTCCTACGGGAGGCAG	194	55	Amann et al., 1990
	Reverse	GTATTACCGCGGCTGCTG		59	
Firmicutes	Forward	TGAAACTAAAGGAATTGACG	135	53	Luu et al., 2017
	Reverse	ACCATGCACCACCTGTC		53	
Bacteroidetes	Forward	GGAACATGTGGTTTAATTCGATG	187	57	Luu et al., 2017
	Reverse	AGCTGACGACAACCATGCAG		57	
Prevotellaceae	Forward	CGAAAGGTTTAGCGGTGAAG	140	57	Song et al., 2020
	Reverse	CGTAGGAGTTTGGACCGTGT		59	
Oscillospira	Forward	ACGGTACCCCTTGAATAAGCC	162	60	Walker et al., 2010
	Reverse	TCCCCGCACACCTAGTATTG		60	
***Clostridium* ASF356**	Forward	GATGCCTCCTAAGAACCGTATGC	151	60	Erny et al., 2015
	Reverse	GCGGACGGGTGAGTAACGT		60	
Ruminococcus	Forward	TTAACACAATAAGTWATCCACCTGG	314	57	Tang et al., 2021
	Reverse	ACCTTCCTCCGTTTTGTCAAC		58	

Results

qPCR analysis confirmed results obtained by metagenomic analysis. Chic diet showed a slight decrease of the F/B ratio due probably to the STL content as STL diet triggered a significant decrease of this ratio (Figure S7_1).

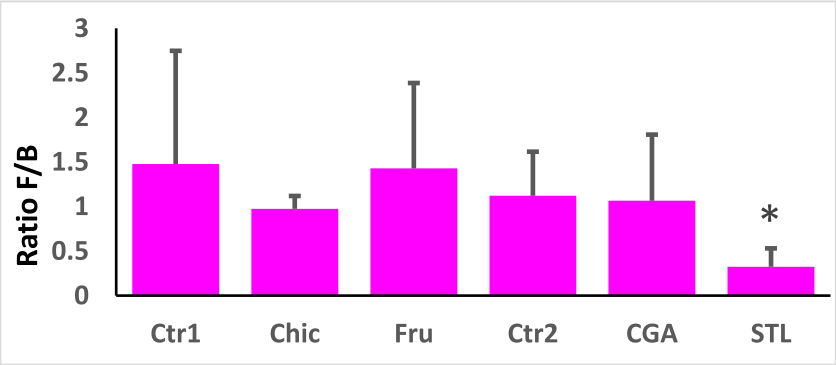

Figure S7_1. Relative abundance of *Firmicutes* and *Bacteroidetes* in mice microbiota after different diets. qPCR results expressed as ratio ΔCt *Firmicutes*/ΔCt *Bacteroidetes*, mean ± SD (Tukey's test, n=5/group, * $p < 0.05$).

The diet-dependent abundance of the four bacterial taxa showed similar tendencies as those observed in metagenomic analysis (Figure S7_2). *Prevotellaceae* and *Clostridium* ASF 356 abundance was found slightly increased during Chic, CGA and STL diets. *Oscillospira* and *Ruminococcus* were found with a decreased abundance during Chic and Fru but also CGA and STL diets. Although Fru and CGA appear to be implicated in microbiota changes, STL were found to play the most pregnant role.

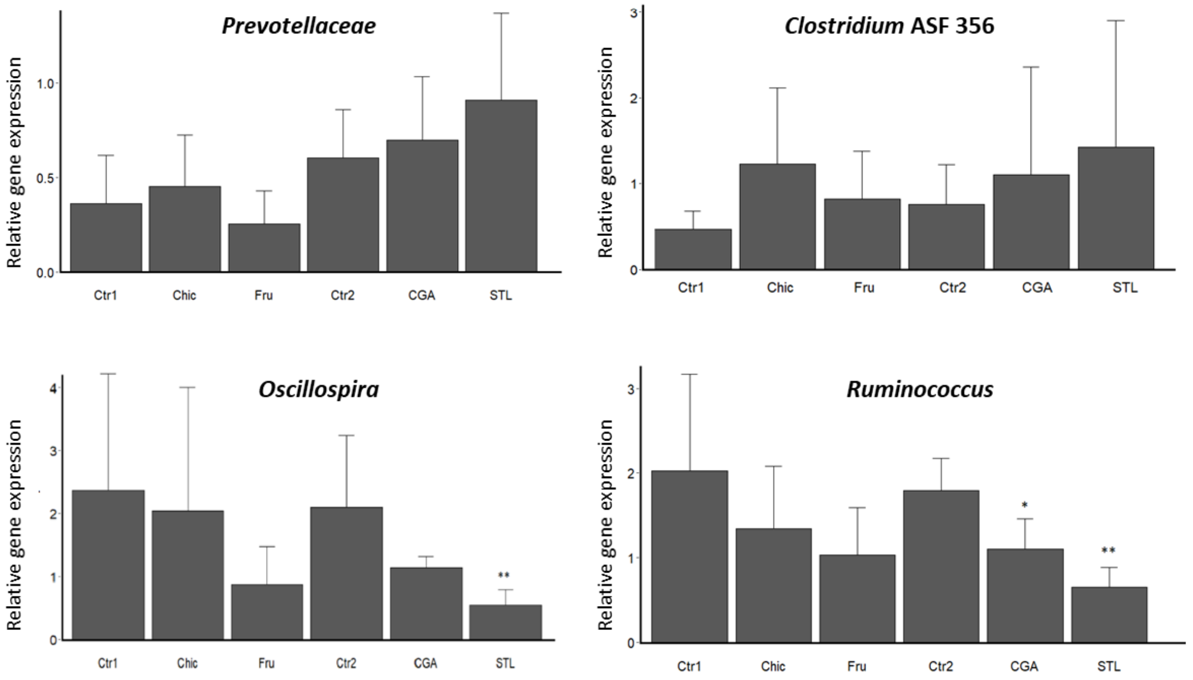

Figure S7_2. Relative abundance of four bacterial taxa from gut microbiota of mice, impacted by chicory, fructose, CGA or STL supplemented diet. qPCR results expressed as the 2^-ΔΔCt mean ± SE (Student's test, n=5/group, * $p < 0.1$, ** $p < 0.05$).

References

Amann RI, Binder BJ, Olson RJ, Chisholm SW, Devereux R, Stahl DA. (1990) Combination of 16S rRNA-targeted oligonucleotide probes with flow cytometry for analyzing mixed microbial populations. Applied and Environmental Microbiology 56(6): 1919-25.

Chaplin A, Parra P, Laraichi S, Serra F, Palou A. (2015) Calcium supplementation modulates gut microbiota in a prebiotic manner in dietary obese mice. Molecular Nutrition and Food Research: 468–480.

Cole, JR, Wang Q, Fish JA, Chai B, McGarrell DM, Sun Y, Brown CT, Porras-Alfaro A, Kuske CR & Tiedje JM. (2014) Ribosomal Database Project: Data and tools for high throughput rRNA analysis. Nucleic Acids Research 42(D1): 633–642.

CorpetF. (1988) "Multiple sequence alignment with hierarchical clustering". Nucleic Acids Research 16(22): 10881-10890.

Livak K & Schmittgen T. (2001) Analysis of Relative Gene Expression Data Using Real-Time Quantitative PCR and the 2−ΔΔCT Method. Methods 25(4): 402-408.

Luu TH, Michel C, Bard JM, Dravet F, Nazih H, Bobin-Dubigeon C. (2017) Intestinal Proportion of Blautia sp. is Associated with Clinical Stage and Histoprognostic Grade in Patients with Early-Stage Breast Cancer. Nutrition and Cancer 69(2): 267-275.

Parnell JA, Reimer RA. (2012) Prebiotic fiber modulation of the gut microbiota improves risk factors for obesity and the metabolic syndrome. Gut Microbes 3(1): 29−34.

Untergasser A, Cutcutache I, Koressaar T, Ye J, Faircloth BC, Remm M & Rozen SG. (2012) Primer3-new capabilities and interfaces. Nucleic Acids Research, 40(15): 1–12.

Song S, Liu J, Zhang F and Hong JS. (2020) Norepinephrine Depleting Toxin DSP-4 and LPS Alter Gut Microbiota and Induce Neurotoxicity in α-Synuclein Mutant Mice. Scientific Reports 10(1): 15054.

Tang S, Cheng Y, Wu T, Hu F, Pan S and Xu X. (2021) Effect of Lactobacillus Plantarum-Fermented Mulberry Pomace on Antioxidant Properties and Fecal Microbial Community. Lebensmittel-Wissenschaft & Technologie 147: 111651.

Erny D, Hrabě de Angelis AL, Jaitin D, Wieghofer P, Staszewski O, David E, Keren-Shaul H and al. (2015) Host microbiota constantly control maturation and function of microglia in the CNS. Nature Neuroscience 18(7): 965-77.

Chuanren D, Cui Y, Zhao Y, Zhai J, Zhang B, Zhang K, Sun Dand Chen H. (2016) Evaluation of Faecalibacterium 16S RDNA Genetic Markers for Accurate Identification of Swine Faecal Waste by Quantitative PCR. Journal of Environmental Management 181: 193-200.

Walker AW, Ince J, Duncan SH, Webster LM, Holtrop G, Ze X, Brown D, Stares MD, Scott P, Bergerat A, Louis P, McIntosh F, Johnstone AM, Lobley GE, Parkhill J & Flint HJ. (2010) Dominant and diet-responsive groups of bacteria within the human colonic microbiota. The ISME Journal 5(2): 220–230.
