# Supplementary figures and images for: Chicory: Understanding the Effects and Effectors of This Functional Food
Source: Nutrients. 2022 Feb 23;14(5):957. doi: 10.3390/nu14050957 (PMC8912540; doi:10.3390/nu14050957)

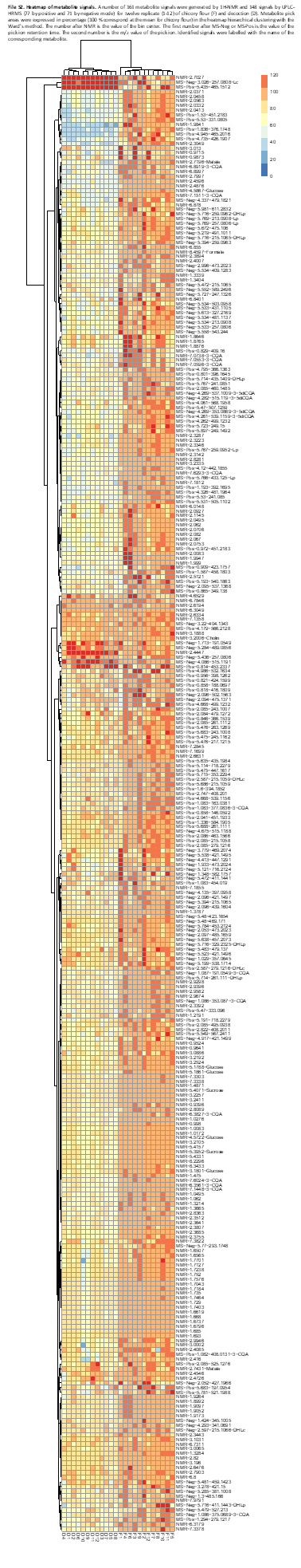

Supplement: Supplementary file 1 [file nutrients-14-00957-s001.zip › File S2.tif]
